# A phosphorus-containing hyperbranched phthalocyanine flame retardant for epoxy resins

**DOI:** 10.1038/s41598-021-96927-y

**Published:** 2021-09-06

**Authors:** Penglun Zheng, Rui Wang, Donghui Wang, Xiaoliang Peng, Yang Zhao, Quanyi Liu

**Affiliations:** 1 College of Civil Aviation Safety Engineering, Civil Aviation Flight University of China, Guanghan, 618307 China; 2 Civil Aircraft Fire Science and Safety Engineering Key Laboratory of Sichuan Province, Deyang, China; 3 School of Vehicle and Mobility, Tsinghua University, Beijing, China

**Keywords:** Polymer characterization, Polymer synthesis

## Abstract

A hyperbranched phosphorus-containing copper phthalocyanine compound (DOPO-CuPc) was successfully synthesized and used as the flame-retardant additive to prepare flame-retarded epoxy thermosets. The addition of DOPO-CuPc led to a significant enhancement of the flame retardant properties of the epoxy resin. The 15DOPO-CuPc/EP composite obtained a LOI value of 35.8%, and the UL-94 rose from NR to V-0 rating. And the addition of DOPO-CuPc resulted in early decomposition of the epoxy thermoset, but the residual char at 700 °C reached 27.7%. The flame retardant mechanism was further investigated. It was found that DOPO-CuPc could release phosphorus-containing radicals and non-combustible gases in the gas phase to exert gas-phase flame retardant activity. In the condensed phase, the epoxy thermoset formed the expanded honeycomb-like char layer during combustion and the presence of copper phthalocyanine contributed to the stability of the char layer.

## Introduction

As a general thermoset, epoxy resins are widely applied in high-performance engineering fields due to its outstanding mechanical strength, excellent adhesive strength, and superior chemical resistance^1,2^. As with other commonly used engineering plastics, high flammability is one of the major drawbacks^3,4^. The flammability of epoxy resins and the resulting fire hazards are a constant threat to people's lives and property, which severely limits their use in applications requiring higher levels of flame retardancy. The addition of halogenated compounds to epoxy resins is a widely used method for preparing flame retardant epoxy resins. Unfortunately, numerous halogenated flame retardants have been listed as persistent organic pollutants (POPs) due to the enormous ecological and human health hazards caused by the products released during combustion^5^. Recently, researchers are interested in finding eco-friendly flame retardants to alternative halogen-based flame retardants^6,7^.

Currently, phosphorus-containing flame retardants are favored among the various flame retardants due to low toxicity and abundant resources. A typical phosphorus-containing flame retardant, 9,10-dihydro-9-oxa-10-phosphaphenanthrene-10-oxide (DOPO), has been proved to be the effective flame retardant for epoxy resins^8^. DOPO exhibit higher thermal and chemical stability than other uncycled organophosphorus compounds due to the biphenyl and heterophilic in structure^9^. In addition, the DOPO structure contains active P–H bonds, which are highly reactive to electron-deficient groups such as olefins, epoxides and carbonyl groups, and can be reacted to generate numerous derivatives^10–12^. However, the flame-retardant efficiency of DOPO-based flame retardants containing only phosphorus is generally unsatisfactory. In order to improve the flame retardancy of DOPO-based flame retardants and to reduce their negative effects on other properties of epoxy resin, it is an effective way to combine phosphorus with other flame retardant elements to form a synergistic flame retardant effect. Shang et al. designed a bio-based epoxy resin (TEBA) containing phosphorus and nitrogen^13^. The results showed that the LOI of TEBA increased by about 64% compared to the original epoxy resin and the UL-94 rating was improved from no rating to V-0. Zhu et al. designed a novel phosphorus-nitrogen containing oligomer (BPOPA) as an additive to enhance the flame retardancy of epoxy resin/ammonium polyphosphate^14^. It was found that the epoxy composites with 7.5 wt% ammonium polyphosphate and 2.5 wt% BPOPA passed UL-94 V-0 test and with an LOI value of 33.1%. And BPOPA exhibited binary flame retardant activity in both gas phase and condensed phase. You et al. reported a new type of phosphorus-nitrogen flame retardant (TNTP) and applied it in epoxy resin^15^. It was found that a 5 wt% loading of TNTP can impart satisfactory flame retardancy and thermal stability to epoxy resins.

Hyperbranched polymers are of increasing interest to polymer scientists because of their unique structural features, synthetic methods, and large number of functional end-groups. In recent years, hyperbranched polymers are widely used as flame retardants for polymers. Phthalocyanines, a hyperbranched organic macromolecular compound with dendritic molecular structure, have attracted attention for their unique two-dimensional co-planar aromatic heterocyclic structure^16,17^. This special conjugate structure imparts exceptional thermal and chemical stability to the phthalocyanine. At the same time, phthalocyanines contain a high content of nitrogen, which shows significant potential for providing flame retardancy and thermal stability to polymers^18^. On the one hand, it provides thermal and chemical stability to the polymer by using phthalocyanine as an additive. On the other hand, phthalocyanines can be used in combination with other flame retardant elements to provide synergistic flame-retarding effect, such as phosphorus-based flame retardants. In addition, the phthalocyanine molecule can be coordinated with numerous metallic elements to form metal phthalocyanines to satisfy the requirements of different engineering fields due to their strong coordination capabilities^19,20^. As far as we know, phthalocyanines have a wide range of applications in various research fields such as solar cells, photoelectric conversion, electrical signal storage, and catalytic materials, but the application in the field of flame retardancy has rarely been reported.

At present, phosphorus–nitrogen synergistic flame retardant modification has been proved to be a very efficient method to reinforce the flame retardancy of epoxy resins. In this research, we present a DOPO-based hyperbranched copper phthalocyanine (DOPO-CuPc) by grafting reaction of DOPO with copper phthalocyanine and introduced it into the epoxy resin as an additive. The structure of DOPO-CuPc was characterized. The flame retardant properties and thermal stability of the modified epoxy thermosets were evaluated by flammability tests, and the flame retardant mechanism of DOPO-CuPc was analyzed.

## Materials and methods

### Materials

DOPO was purchased from Adamas-beta^®^. The Bisphenol A type phthalonitrile-based resin containing benzoxazine (BAPh) was purchased from Chengdu Dymatic Fine Chemicals Co., Ltd. Anhydrous cuprous chloride (CuCl) was purchased from Xilong Chemical Co., Ltd. N, N-Dimethylacetamide (DMAc), Methanol and Tetrahydrofuran (THF) were purchased from Chengdu Kelon Chemical Co., Ltd. The 4, 4’-diaminodiphenylsulphone (DDS) was purchased from Wenzhou Shoucheng Chemical Technology Co., Ltd. Diglycidyl ether of bisphenol A (DGEBA, E-51, epoxide value of 0.51 mol/100 g) was obtained from Shandong Jiaying Chemical Technology Co., Ltd., China.

### Synthesis of DOPO-CuPc

DOPO (21.6 g), BAPh (36.9 g) and THF (200 ml) were mixed in a 500 ml four-necked round-bottom flask equipped with a stirrer and a reflux condenser. After stirring at room temperature for 0.5 h, the mixture was reacted under nitrogen protection at 80 °C for 12 h. The solvent was removed using a rotary evaporator, washed with water and dried under vacuum at 60 °C for 12 h. Finally, DOPO-BAPh was obtained.

**Scheme 1:**
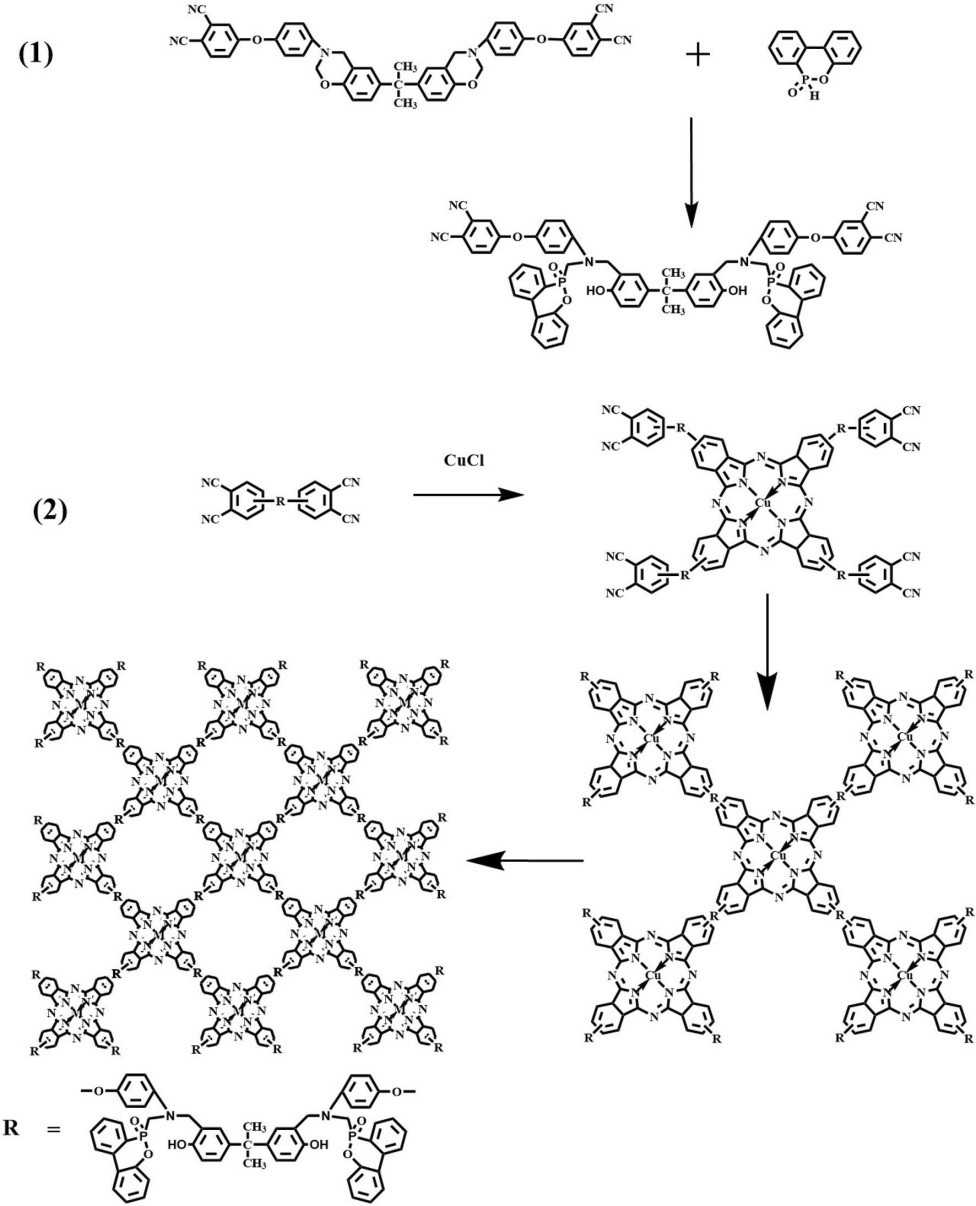
Synthetic route of DOPO-CuPc.

DMAc (200 ml) was added to a 500 ml four-necked round-bottom flask equipped with a stirrer and reflux condenser. Then Bisphenol A type phthalonitrile-based resin containing benzoxazine (3.52 g) and CuCl (0.1 g) were added to the flask, stirred until fully dissolved, and the reaction was kept at 160 °C for 4 h. The product resulting from the reaction was washed, filtered, and dried at 80 °C. The obtained product was then filtered by using methanol at 80 °C and room temperature several times, respectively, and then dried at 70 °C for 12 h in vacuum oven. The final green powder obtained was the target product (DOPO-CuPc) (Scheme 1).

### Preparation of samples

The modified epoxy thermoset resin was prepared as follow: first, the epoxy resin was placed in an oven at 120 °C for 15 min, and DDS was added to the epoxy resin and stirred until well-dispersed. Afterwards, DOPO-CuPc was added to the resulting mixture with strong stirring. Finally, the mixture was rapidly poured into the preheated mold and cured at 190 °C for 4 h. In addition, it was noted that the air bubbles in the mixture should be removed using a vacuum oven before curing. The neat epoxy resin was prepared according to this procedure, except for the absence of DOPO-CuPc. The detailed composition was shown in Table 1.

**Table 1:** Formulations of epoxy thermosets.

Sample	Composition (phr)			Content of DOPO-CuPc (wt%)
	Cured EPs	DDS	DOPO-CuPc	
Neat EP	100	27	0	0
5DOPO-CuPc/EP	100	27	5	3.79
10DOPO-CuPc/EP	100	27	10	7.29
15DOPO-CuPc/EP	100	27	15	10.6
20DOPO-CuPc/EP	100	27	20	13.6

### Characterization

The Fourier-transform infrared (FTIR) were conducted on a Perkin-Elmer Spectrum Two FTIR spectrometer. The test was performed by using the KBr pellets method. The detailed conditions are as follows: the spectral resolution was 4/cm, the number of scans was 32, the scan wave number range was 4000–640/cm.

The flammability of modified epoxy thermosets was evaluated using limiting oxygen index (LOI) and UL-94. The value of LOI was acquired by using a JF-3 oxygen index meter (Nanjing Jiangning Analytical Instrument Co., Ltd.) according to ASTM D2863. The dimensions of the samples were 130 mm × 6.5 mm × 3 mm. The vertical burning test were determined by using a CZF-3 burning tester (Nanjing Jiangning Analytical Instrument Co., Ltd.) according to ASTM D3801. The dimensions of the samples were 130 mm × 12.7 mm × 3 mm.

The combustion behaviors of the modified epoxy thermosets were recorded using a cone calorimeter (Kunshan Modisco Combustion Technology Instrument Co., Ltd.) according to ISO 5660-1 at a heat flux of 50 kW/m^2^. And the dimensions of the samples were 100 mm × 100 mm × 3 mm.

Thermogravimetric analyses (TGA) of the modified epoxy thermosets were con-ducted from a Pyris thermogravimetric analyzer (Perkin-Elmer TGA 4000) under nitrogen atmosphere with gas flow of 20 ml/min. A sample with a weight of about 10 mg was used and heated from 30 to 800 °C at a rate of 10 °C/min. In addition, the samples were dried before the test started.

The thermogravimetry-infrared spectroscopy (TG-IR) was studied by connecting a TGA 4000 thermogravimetric analyzer and a Spectrum Two FTIR spectrophotometer through a special tube for transferring the gas. The test conditions of TG-IR are the same as the TGA.

The residual chars of the modified epoxy thermosets after cone calorimetric test were carried out using a scanning electron microscope (SEM) (Hitachi S-4800) with an accelerating voltage of 3 kV. Samples were sprayed with gold before the test started.

## Results and discussion

### Structural characterizations of DOPO-CuPc

**Figure 1:**
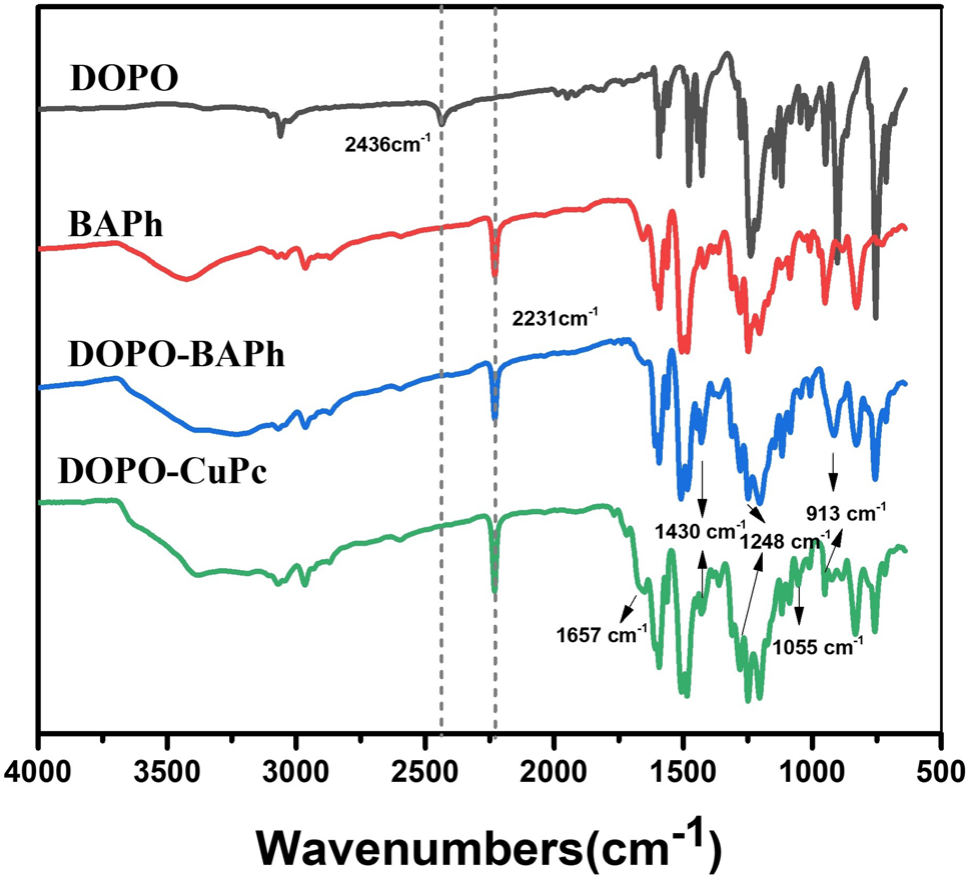
FTIR spectrum of DOPO, BAPh, DOPO-BAPh and DOPO-CuPc.

The infrared spectra of DOPO, BAPh, DOPO-BAPh and DOPO-CuPc were shown in Fig. 1. From the curves of DOPO, BAPh and DOPO-BAPh, the P–H bonds at 2436/cm was disappeared in DOPO-BAPh, and the remain of the nitrile groups (2231/cm), P=O bonds (1248/cm), P–O–Ph bonds (913/cm), P–Ph bonds (1430/cm) indicated that the DOPO-BAPh was successfully synthesized. In addition, the new appeared peaks at 1055/cm and 1657/cm were attributed to the metal–ligand (Cu–N) vibration^21^ and phthalocyanine. And the nitrile groups, P-containing bonds were still detected in DOPO-CuPc, based on the analysis above, the DOPO-CuPc was successfully prepared as designed.

### Thermal analysis of epoxy thermosets

**Figure 2:**
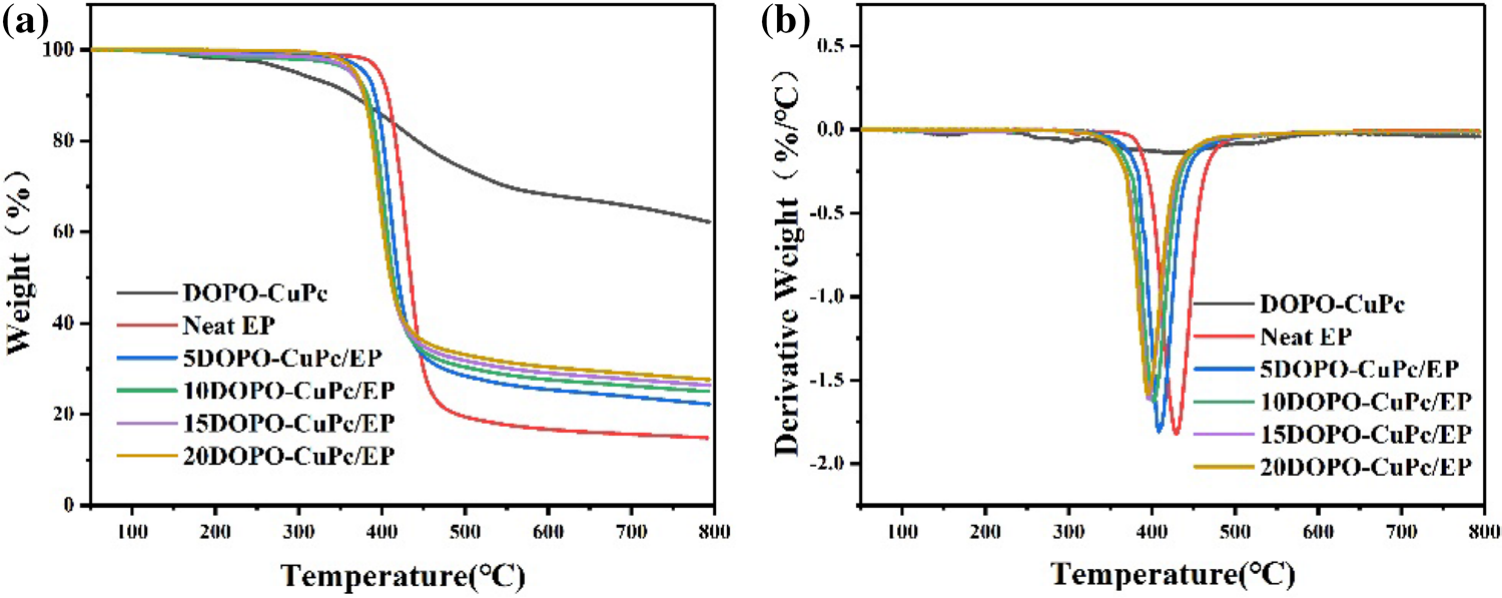
(**a**) TGA and (**b**) DTG curves of DOPO-CuPc and epoxy thermosets under N_2_ atmosphere.

The thermal stability of the modified epoxy thermosets was studied by TG and derivative thermogravimetric (DTG) in nitrogen. Figure 2 showed the TG and DTG curves of the modified epoxy thermosets in nitrogen. The 5% weight loss temperature (T_5%_), maximum rate of weight loss (V_max_), maximum temperature of weight loss (T_max_) and weight of char yield at 700 °C (C_y700_) were listed in Table 2. From the TG curves it could be noted that all samples displayed a major thermal weight loss phase. However, after the addition of DOPO-CuPc, the initial decomposition temperature of the epoxy thermosets decreased with the increase of phosphorus content in the early degradation stage. It was attributed to the introduction of O=P–O bonds with low thermal stability in the epoxy resin. The 5% weight loss temperature and maximum temperature of weight loss of 10DOPO-CuPc/EP composite were reduced by 33.2 °C and 27.2 °C, respectively compared to the original epoxy resin. Although the addition of DOPO-CuPc resulted in a lower initial decomposition temperature of the epoxy thermosets, it was still higher than 350 °C and maintained a relatively stable thermal stability. Moreover, the char yield of the epoxy thermosets at 700 °C increased with the addition of DOPO-CuPc, which increased from 15.6 to 26.2% with only 10% addition of COPO-CuPc. In addition, the DTG curves showed that the decomposition rate of the epoxy thermosets decreased with increasing phosphorus content, which revealed the inhibition of thermal degradation by flame retardant epoxy resin.

**Table 2:** TGA Data of epoxy thermosets under nitrogen.

Sample	T_*5%*_ (°C)	T_*max*_ (°C)	V_*max*_ (°C)	C_*y700*_ (%)
DOPO-CuPc	297.6	413.9	1.42	68.0
Neat EP	397.3	428.5	1.82	15.6
5DOPO-CuPc/EP	379.6	408.4	1.81	23.8
10DOPO-CuPc/EP	364.1	401.3	1.62	26.2
15DOPO-CuPc/EP	363.2	396.4	1.61	27.7
20DOPO-CuPc/EP	368.1	394.9	1.58	28.9

### Flame retardancy of epoxy thermosets

The flame retardant properties of DOPO-CuPc/EP composites were analyzed using LOI and UL-94 vertical burning tests. The results of the tests were presented in Table 3. The neat epoxy resin showed inherent flammability with an LOI of only 22.0%, and in the UL-94 tests, the sample burned continuously after ignition and with molten drip-ping, failing to achieve any flame resistance level. The flame retardant properties of epoxy thermosets were enhanced considerably with the incorporation of DOPO-CuPc. When the addition of DOPO-CuPc was 5 wt%, the LOI value increased to 29.0% and reached UL-94 V-1 rating with no dripping and the total burning time (t_1_ + t_2_) was reduced to 26 s. With a further increase in the DOPO-CuPc content to 15 wt%, the modified epoxy thermosets achieved V-0 rating and the LOI value increased to 35.0%, and t_1_ + t_2_ reduced to 9 s. These results indicated that DOPO-CuPc possessed excellent flame retardant properties.

**Table 3:** Flame retardant properties of epoxy thermosets.

Samples	LOI (%)	UL-94		
		t_1_ + t_2_ (s)	Dripping	Rating
Neat EP	21.7	> 100	Yes	NR
5DOPO-CuPc/EP	30.0	12 + 14	No	V-1
10DOPO-CuPc/EP	33.0	6 + 7	No	V-1
15DOPO-CuPc/EP	35.8	4 + 5	No	V-0
20DOPO-CuPc/EP	37.2	4 + 3	No	V-0

**Figure 3:**
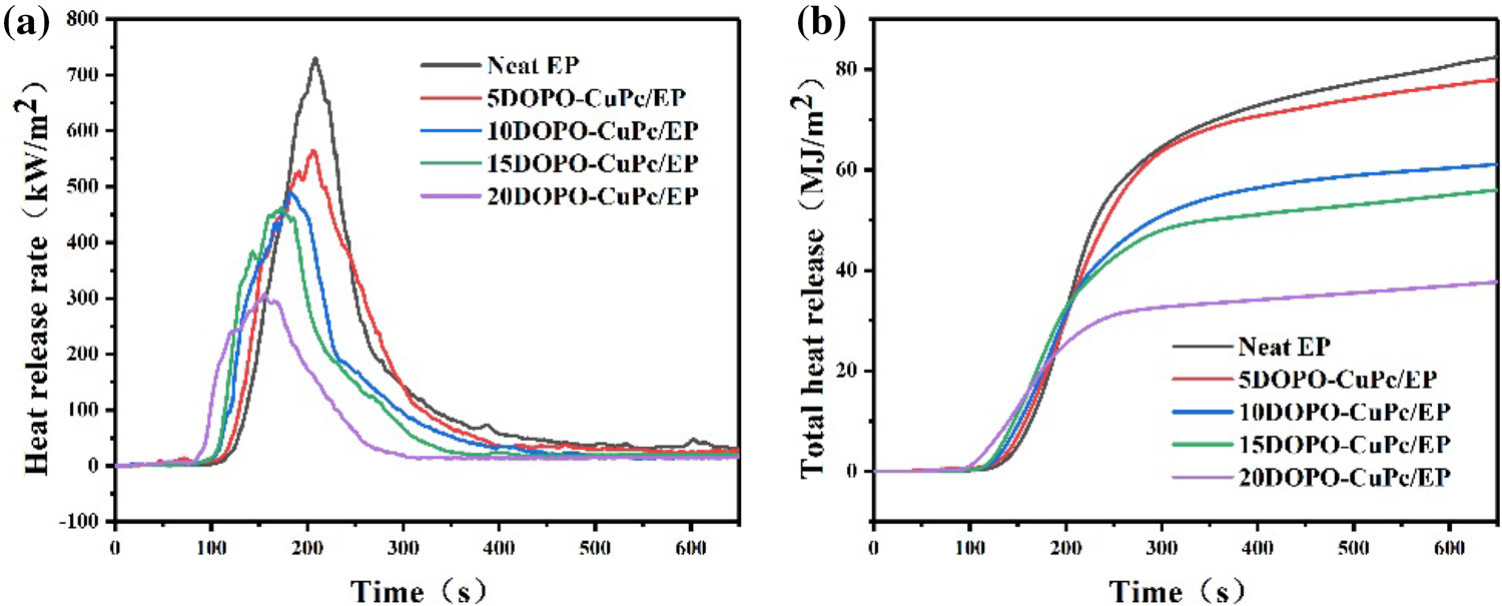
HRR (**a**) and THR (**b**) curves of epoxy thermosets.

To further investigated the flame retardancy of epoxy thermosets, cone calorimeter tests were conducted. The crucial parameters such as the time to ignition (TTI), heat release rate (HRR), peak of HRR (PHRR), total heat release (THR) and total mass loss (TML) were obtained by cone calorimeter. The results were presented in Fig. 3 and Table 4. It can be seen from the TTI in Table 4 that flame-retardant epoxy resins ignited earlier than neat epoxy resins. It was attributed to the early decomposition of DOPO-CuPc in the low temperature region while promoting the early pyrolysis of the epoxy resin matrix. As shown in Fig. 3, from the HRR curve of neat EP, it could be indicated that the HRR value rose fast and reached a PHRR value of 729.8 kW/m^2^, and the introduction of DOPO-CuPc made the PHRR of epoxy thermosets lower and appear earlier. The PHRR of 5DOPO-CuPc/EP composites was reduced to 565 kW/m^2^. The continued addition of DOPO-CuPc led to a further decrease in PHRR. The PHRR of 20DOPO-CuPc/EP composites was reduced to 306.9 kW/m^2^, which was 57.9% lower compared to the neat epoxy resin. The THR of the neat epoxy resin was 88.4 MJ/m^2^. The THR of DOPO-CuPc/EP composites was reduced by 57.3% compared with that of neat epoxy resin. The results indicated that DOPO-CuPc inhibited the thermal decomposition of epoxy thermosets. In addition, the mass loss rate of the epoxy cured products was reduced from 75.8 to 59.4% with the introduction of DOPO-CuPc. It implied that more residual char was generated during combustion, which inhibited the combustion of the epoxy thermosets and prevented the heat transfer to the matrix interior, proved the flame retardant effect in the condensed phase.

**Table 4:** Fire parameter of epoxy thermosets.

Sample	TTI (s)	PHRR (kW/m^2^)	THR (MJ/m^2^)	TML (wt%)
Neat EP	63	729.8	88.4	75.8
5DOPO-CuPc/EP	50	565.0	77.6	70.5
10DOPO-CuPc/EP	46	491.1	61.1	68.8
15DOPO-CuPc/EP	43	461.0	55.9	64.3
20DOPO-CuPc/EP	38	306.9	37.7	59.4

**Figure 4:**
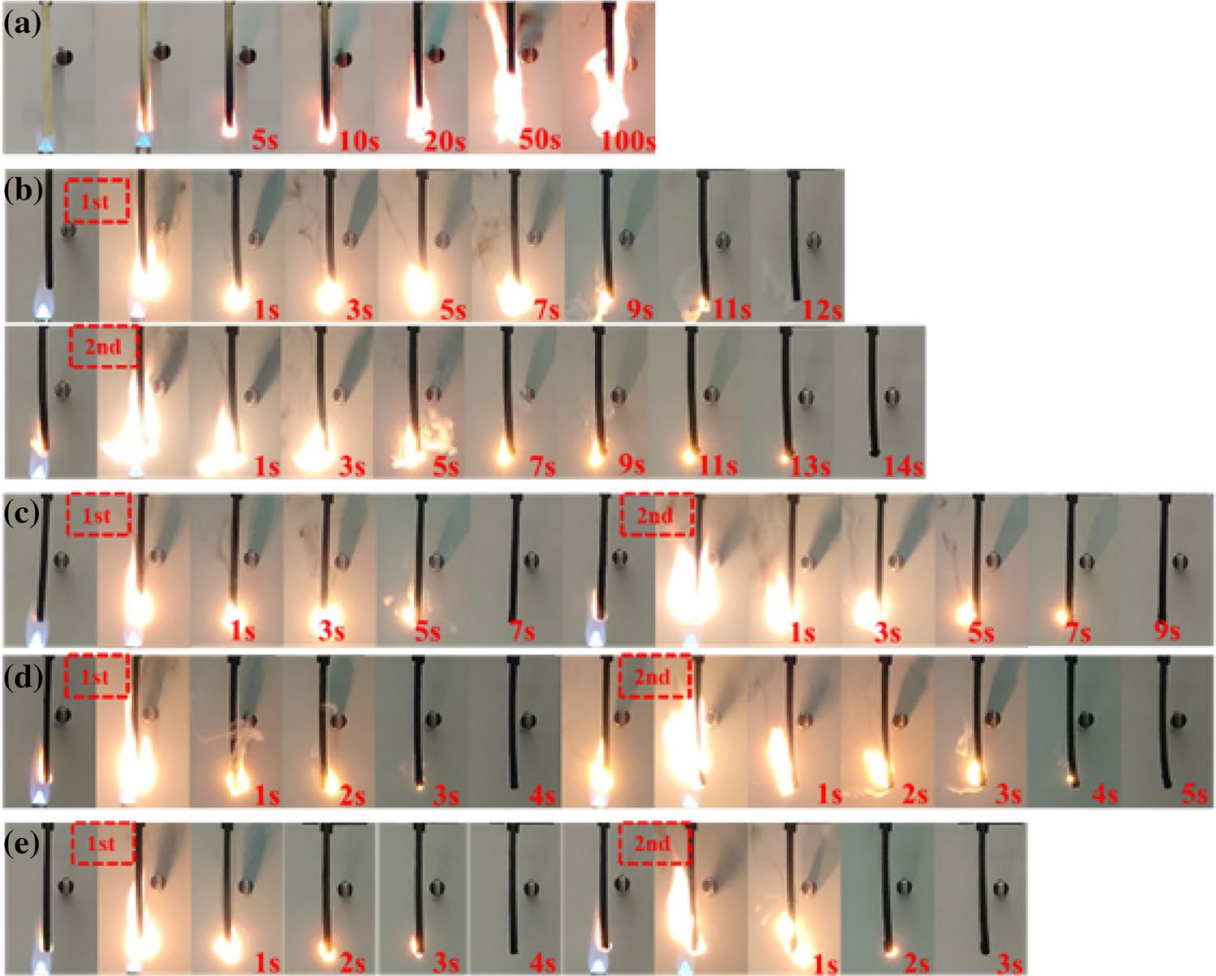
Digital photographs of epoxy thermosets during UL-94 testing. (**a** EP, **b** 5DOPO-CuPc/EP, **c** 10DOPO-CuPc/EP, **d** 15DOPO-CuPc/EP, **e** 20DOPO-CuPc/EP).

In order to better investigated the effect of DOPO-CuPc on the flame retardancy of epoxy resin, the combustion process of epoxy thermosets in UL-94 test was recorded by a digital camera. Figure 4 presented the screenshots of the video during the UL-94 test. The neat epoxy resin burned vigorously with dripping after ignition and continued to burn for more than 100 s. With the introduction of DOPO-CuPc, the combustion behaviour of epoxy thermosets changed significantly, as reflected by the ability to self-extinguish rapidly and without dripping. Furthermore, it was noticed that the gases generated during combustion were blown out from the inside of the samples. Yang et al. had observed this phenomenon in their research and named it as “blowing-out effect”^22^. The blowing-out effect might contribute to flame retardancy of modified epoxy thermosets.

**Figure 5:**
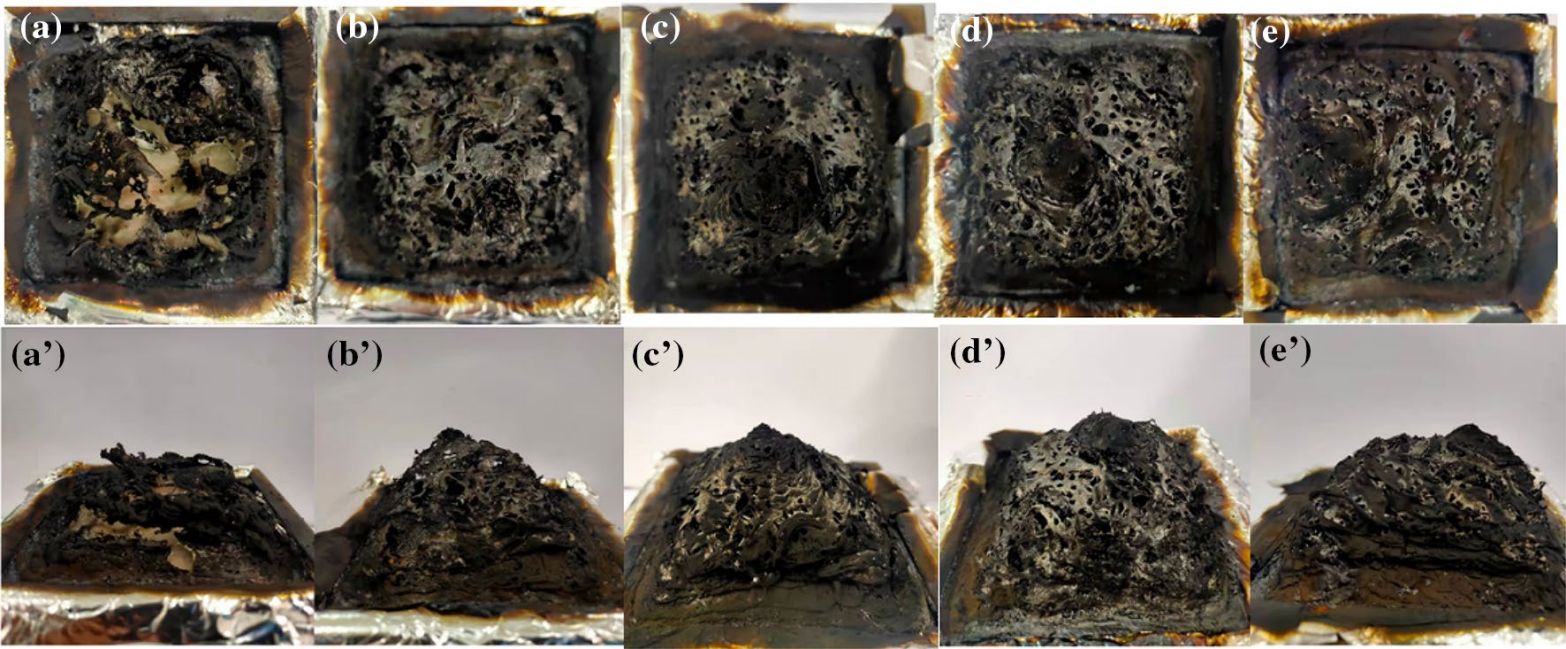
Video screenshots of char residues from cone calorimeter tests of (**a** and **a′**) EP, (**b** and **b′**) 5DOPO-CuPc/EP, (**c** and **c′**) 10DOPO-CuPc/EP, (**d** and **d′**), 15DOPO-CuPc/EP, (**e** and **e′**) 20DOPO-CuPc/EP.

The analysis of the residual char formed after the combustion of epoxy thermosets could provide a lot of information which reflect the flame retardant mechanism in the condensed phase. The digital photographs of the residual char of the modified epoxy thermoset resin after cone testing were presented in Fig. 5. The original epoxy resin remained only a minor amount of residual char after cone calorimeter tests, and the tinfoil at the bottom was burned through. The amount of residual char after combustion of epoxy thermosets increased significantly with the addition of DOPO-CuPc, which indicated the superior catalytic char formation ability of DOPO-CuPc. In addition, the presence of a significant number of benzene rings in the structure of DOPO-CuPc contributed to the formation of the stable char layer during combustion. Moreover, the residual char of the modified epoxy thermosets exhibited the swollen honeycomb-like char layer. The expanded dense residual char in the internal honeycomb cavity effectively isolated heat and oxygen. Further, some small molecules of gas generated during the combustion was continuously contained by the char layer. The contained gases accumulated until the char layer broke down and blew out immediately, which exhibited the “blowing-out effect”.

### Microscopic morphologies of char residues

**Figure 6:**
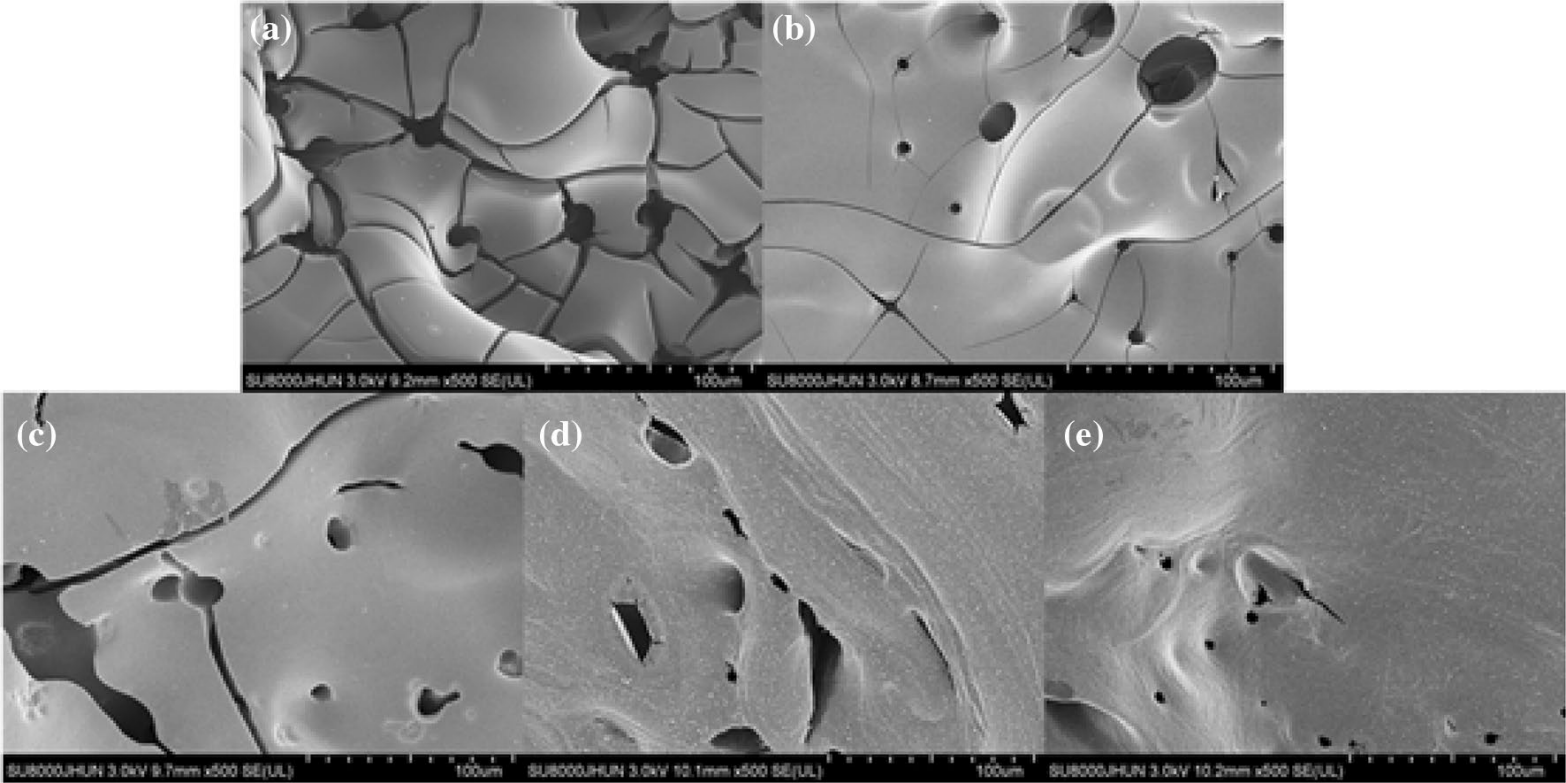
SEM micrographs (**a** neat epoxy resin, **b** 5DOPO-CuPc/EP, **c** 10DOPO-CuPc/EP, **d** 15DOPO-CuPc/EP, **e** 20DOPO-CuPc/EP) of residue chars of epoxy thermosets after cone test.

The residual char of the epoxy thermosets after cone calorimeter testing was collected for SEM to further analysed the microstructure of the surface of residual char. As shown in Fig. 6, the SEM image of neat epoxy resin showed a significant number of cracks and pores in the residual char. The broken char layer was unable to prevent the transfer of oxygen and heat during the combustion. For DOPO-CuPc/EP composites, the char layer after combustion showed a relatively complete structure with smaller cracks and pores and flattered with increasing amount of DOPO-CuPc addition. On the one hand, the phosphorus-containing groups contributed to the char-forming ability; On the other hand, the cross-linked net-like structure of phthalocyanine reinforced the strength of the residual char. As shown in Fig. 6e, the char layer of 20DOPO-CuPc/EP composite exhibited a uniform and crack-free morphology, which protected the underlying epoxy resin matrix substrate from thermal radiation, thereby improving the flame retardant properties of epoxy resin^23^.

### TG-IR analysis of EP composites

**Figure 7:**
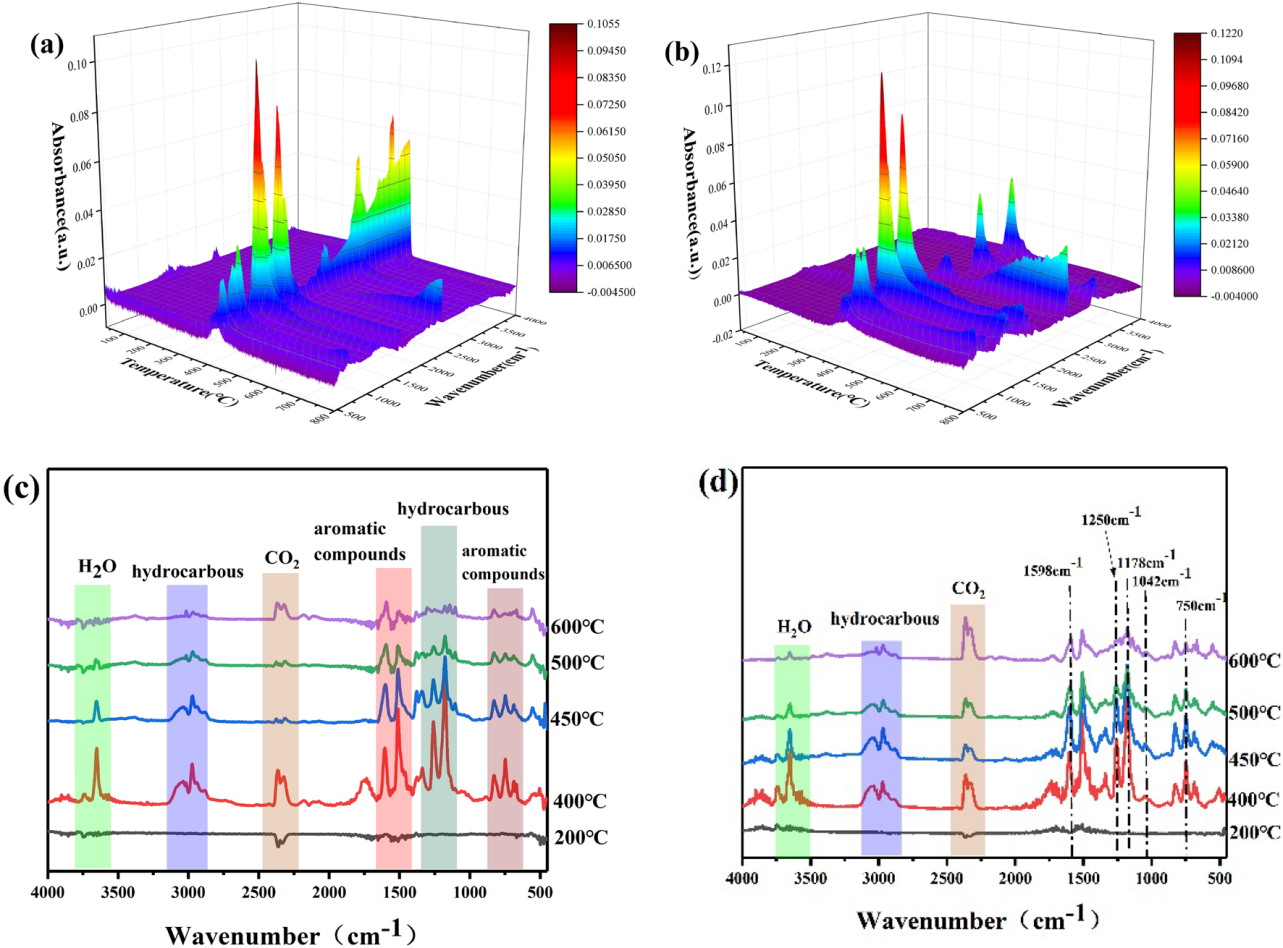
Three-dimensional spectra of epoxy thermosets pyrolysis products and IR spectra of pyrolysis products at different temperatures. (**a**, **c**) EP and (**b**, **d**) 20DOPO-CuPc/EP.

The pyrolysis products of epoxy thermosets were investigated by using TG-IR to elucidate the flame retardant mechanism of DOPO-CuPc in the gas phase. The three-dimensional view of the products generated during the decomposition of the epoxy thermosets and infrared spectra at different temperatures were presented in Fig. 7. Several exemplary products such as hydrocarbons (1100–1300/cm and 2800–3200/cm), CO_2_ (2359/cm), hydroxyl-containing compounds (3500–3700/cm) and aromatic compounds (749/cm, 830/cm, 1508/cm and 1598/cm) were detected in the infrared spectra of neat epoxy resin. There were no emerging peaks from the infrared spectra of 20DOPO-CuPc/EP composites, while the ratio of peaks changed. This phenomenon was attributed to the overlap of the newly emerged peaks with the original peaks. Among them, the peaks at 1250/cm (P=O) and 1598/cm (P–O–Ph) were overlapped with the aromatic compounds (C_Ar_–H and C_Ar_–O), respectively^24,25^. It indicated that the products of polyphosphate structures were generated in the degradation of DOPO-CuPc, which further reacted with other products to form P–O–Ph bond-containing compounds. Meanwhile, the intensity changing of the absorption peak at 749/cm was the contribution of the DOPO group in DOPO-CuPc. The above results indicated that the DOPO-CuPc/EP composite generated P–O–Ph bond-containing polyphosphate compounds during the degradation.

### Mechanism of flame retardancy

**Scheme 2:**
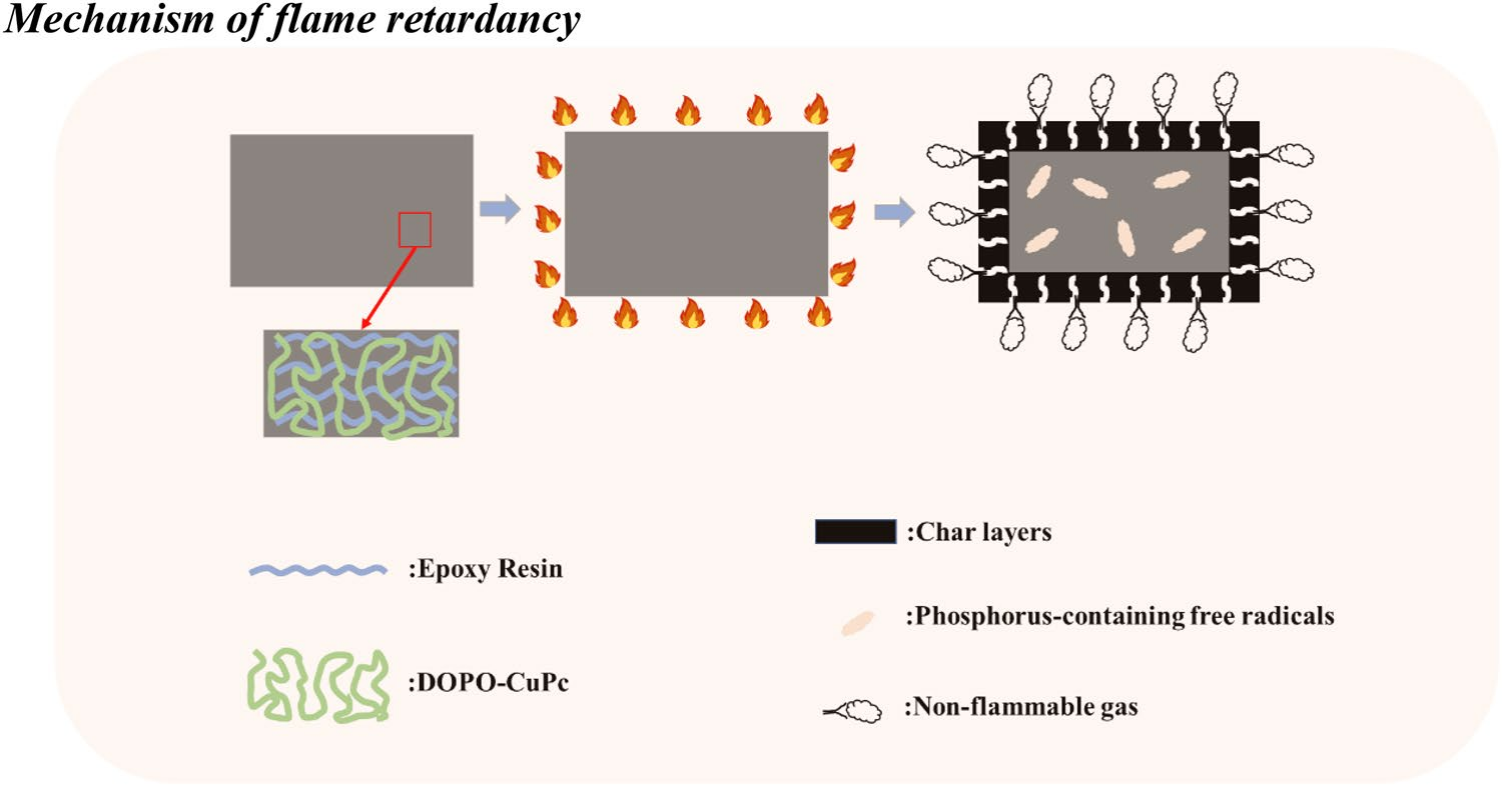
Flame retardant mechanism of DOPO-CuPc.

Based on the above analysis, the flame retardant mechanism of DOPO-CuPc is attributed to the flame-retarding effect of gas phase and condensed phase. The flame retardant mechanism of DOPO-CuPc is shown in Scheme 2. The modified epoxy thermosets generated phosphorus-containing radicals in the thermal decomposition, which could suppress the free radical chain reaction of combustion. And the non-combustible gas produced in the thermal pyrolysis could dilute the combustible gas, thereby inhibiting the continuation of combustion. In addition, the “blowing-out effect” plays an important role in flame retardation in the gas phase. Furthermore, as shown in Table 5, the energy-dispersive X-ray (EDX) tests of the residue char from 20DOPO-CuPc/EP after cone calorimetry test, part of the phosphorus-containing debris remains in the condensed phase, which facilitate the formation of expanded and dense honeycomb-like char layers through dehydration reaction, and the copper element could promote the char-formation during the decomposition. This conclusion has been confirmed by Wang et al.^26^. These char layers effectively act as heat and oxygen barriers, thus enhancing the flame retardancy of the epoxy resin. Besides, the special cross-linked network structure of copper phthalocyanine result in more aromatic structure in the residual char, which enhances the stability of the char layer. In conclusion, DOPO-CuPc is capable of exerting flame-retarding activity in both the gas and condensed phases.

**Table 5:** EDX results of the residue char of 20DOPO-CuPc/EP after cone calorimetry test.

Element	C	N	O	S	P	Cu
Weight content (%)	35.58	12.39	39.25	9.41	2.58	0.79

## Conclusion

A DOPO-based hyperbranched phthalocyanine flame retardant for epoxy resin was obtained via a grafting reaction between DOPO and copper phthalocyanine. Owing to the lower decomposition temperature of DOPO-CuPc, it led to the early decomposition of epoxy resin, but the residual char was enhanced considerably. Besides, the modified epoxy thermosets passed the UL-94 V-0 rating with the LOI value of 35.8% in the loading of 15 wt%. The enhanced flame retardancy of epoxy resins was attributed to the flame retardant activity of DOPO-CuPc in the gas and condensed phases. On the one hand, the generated phosphorus-containing radicals inhibit the combustion process and the non-combustible gas produced dilutes the oxygen concentration. And the blowing-out effect also contributes to the flame retardant activity. On the other hand, the char layer formed by dehydration reaction during combustion acted as a protective char layer in the condensed phase. Furthermore, the presence of copper phthalocyanine reinforces the stability of the char layer.
